# Oral health-related quality-of-life scores differ by socioeconomic status, mother’s level of education, dental visits and severity of malocclusion in mixed dentition of eight-to-ten-year-old schoolchildren

**DOI:** 10.7717/peerj.12062

**Published:** 2021-09-01

**Authors:** Alvaro García Pérez, Álvaro Edgar González-Aragón Pineda, Hilda Gonzalez Olivares

**Affiliations:** 1 Faculty of Higher Studies (FES) Iztacala, National Autonomous University of Mexico (UNAM), Mexico, Mexico; 2 Master’s and Doctoral Program in Medical, Dental and Health Sciences, National Autonomous University of Mexico (UNAM), Mexico City, Mexico

**Keywords:** Socioeconomic factors, Mother’s level of education, OHRQoL, Schoolchildren, Malocclusion

## Abstract

**Objective:**

To determinate the association among socioeconomic status subject’s mother’s level of educational attainment, dental visits, and malocclusion in mixed dentition with on the OHRQoL of eight-to-ten-year-old children.

**Methods:**

A cross-sectional study conducted, in 2019, on Mexican children from households of different socioeconomic status (SES). The prevalence of malocclusion was evaluated using the Dental Aesthetic Index (DAI), while the SES of the participants’ households was evaluated using the three categories (corresponding to a high, middle, or low-income household) stipulated by the *Consejo Nacional de Población* (CONAPO or National Population Council). Oral Health-related Quality of Life (OHRQoL) was evaluated using the Child Perceptions Questionnaire (CPQ_8-10_). Poisson regression models were performed for the analysis of the data obtained.

**Results:**

A total of 79.4% of the subjects presented some type of malocclusion in mixed dentition, which was, by severity, as follows: definite (31.3%); severe (25.6%); and, very severe (22.5%). The Poisson regression model revealed a greater negative impact on the following four CPQ_8-10_domains for children with severe/very severe malocclusion [RR]: oral symptoms [2.78]; functional limitations [2.72]; emotional well-being [2.59]; and, social well-being [3.99]. A greater impact on the four CPQ_8-10_domains was found for children from a low-income household than for children from a high-income (*p* < 0.001) household. Furthermore, poor oral hygiene, lack of dental visits, and the mother’s level of educational attainment (<9 years) were found to have a negative impact on OHRQoL.

**Conclusion:**

The findings of the present study demonstrated that the severity of malocclusion was associated with a greater negative impact on the OHRQoL of children, while those children who face greater health inequalities are likely to report a greater negative impact on their OHRQoL.

## Introduction

Malocclusion is one of the most common dental disorders on a global level, in both the child and adult populations (Zhou et al., 2017). Defined as an alteration in craniofacial growth and development that impedes the correct function of the stomatognathic system, malocclusion may also affect aesthetics and have a psychosocial impact on the lives of both children and adolescents (Martins-Júnior, Marques & Ramos-Jorge, 2012; Jha et al., 2014). Presenting a multifactorial aetiology, malocclusion can occur due to either hereditary or environmental factors, or a combination thereof, which are among those that greatly contribute to the development of dental disease (Zou et al., 2018).

Throughout the mixed dentition period, many changes present that may determine the subsequent normality of the occlusion that develops as a result. The process of transition from temporary to permanent teeth is complex and involves dental eruption, occlusal changes, physiologic spacing, and changes to the dimensions of the dental arches (Piassi et al., 2019). Malocclusions may present during these physiological changes in the mixed dentition and may have a negative impact on children’s self-image (Dimberg, 2015).

Epidemiological studies show a prevalence of malocclusion in mixed dentition ranging from 26.2% to 94% (Tausche, Luck & Harzer, 2004; Yu et al., 2019; Góis et al., 2012). Moreover, it has been reported that, during the mixed dentition phase, 72.7% of children presenting normal occlusion in primary dentition may develop malocclusion in permanent dentition (Legovic & Mady, 1999).

Therefore, malocclusions can occur at any age, which has repercussions on quality of life (Abreu, 2018). The Oral Health-related Quality of Life (OHRQoL)is a multidimensional concept that includes a subjective evaluation of the individual’s oral health in the performance of their daily activities, their functional and emotional well-being, their expectations, and their level of satisfaction (Bennadi & Reddy, 2013). Different studies have shown that oral conditions have a negative impact on quality of life (García-Pérez et al., 2017; Aguilar-Díaz, Irigoyen-Camacho & Borges-Yáñez, 2011). A meta-analysis conducted on the impact of malocclusion on OHRQoL in adolescents found that, the more serious the malocclusion, the greater the negative impact on some of the physical and psychosocial domains of the OHRQoL (Sun, Wong & McGrath, 2017). It should be noted that malocclusion can also present during the mixed dentition phase and can affect the child’s self-image.

Given that appearance is fundamental to social relationships, with children beginning to show their emotions at an early age, there is evidence that children with visible dental differences are likely to be the subject of negative comments and ridicule from their classmates about their appearance (Seehra et al., 2011).

In addition to the presence of malocclusion, other factors have been related to OHRQoL, among them the education of parents and lack of visits to dentist. Parents play an important role in the emotional support and in financing health costs, therefore, parents must be informed and made aware to help their children in the acquisition of behaviors to improve oral health habits and long-term improve your OHRQoL (Naidu & Nunn, 2020).

On the other hand, very few studies report a negative impact of both socioeconomic status (SES) and the presence of malocclusion on OHRQoL (Vedovello et al., 2016; Ravaghi et al., 2019), as SES may exert a modifying effect on the association between clinical conditions and health-related quality of life (Ravaghi et al., 2019), Since children with low economic income present greater social inequalities, including the lack of access to health services. The present study was undertaken in the context of both the importance of conducting OHRQoL studies on eight-to-ten-year-old children and the lack of research on the impact of SES and malocclusion in mixed dentition on the OHRQoL of children of this age. In addition, it is necessary to know what other factors are related between the presence of malocclusion and OHRQoL. The present study aimed to determinate the association among SES, the mother’s level of educational attainment, dental visits, and malocclusion in mixed dentition on the OHRQoL of eight-to-ten-year-old Mexican children. The study also aimed to ascertain whether any differences in OHRQoL are reported based on both the severity of the malocclusion and the SES of the household. The hypothesis proposed in the present study is that those children with severe and very severe malocclusion and living in a household with a low SES experience a greater negative impact on their OHRQoL.

## Material and Methods

A cross-sectional study was conducted, in 2019, on children from households of different SES in Mexico City. The research protocol was reviewed and approved by the Ethics Committee of the Faculty of Higher Studies (FES) Iztacala at the Universidad Nacional Autónoma de México (UNAM or National Autonomous University of Mexico) (CE/FESI/032019/1287). Both the leadership team of the primary schools sampled and the participants’ parents were informed of the protocol, with those parents who agreed to the participation of their children signing the informed consent form.

According to the *Instituto Nacional de Estadística y Geografía* (INEGI or National Institute of Statistics and Geography), the area sampled comprised a population of 360,265 inhabitants (4.1% of the total population of Mexico City), with 77.3% of its households having access to potable water and 98.7% connected to the sewerage system. Moreover, 51.0% of its inhabitants aged 15 and older had been educated up to basic level and 58.7% had access to health services (INEGI, 2015).

The *inclusion criteria* applied for the study were as follows: eight-to-ten-year-old children of either gender; written authorization to participate in the study; the four upper and lower incisors and the first four permanent molars fully erupted; and the parents/guardians of the participant residing at the same address. The *exclusion criteria* applied were as follows: failure to cooperate during the application of the OHRQoL questionnaire; the presence of a dental anomaly (tooth number anomaly); the presence of a craniofacial deformity; a history of dental trauma; and a history of orthodontic treatment. The *elimination criterion* applied corresponded to a failure to cooperate during the physical examination. Based on the exclusion criteria, 30 participants chose not to participate in the study and 25 participants were eliminated from participating.

### Variables

#### Explanatory variables

The following independent variables were applied by the present study: age; sex (boy/girl); SES; toothbrushing frequency (number of times a day) dichotomized into <2 or ≥ 2 times a day; the OHI-S has two components, the Debris Index and the Calculus Index, each of which scores the respective amount of debris or calculus found on pre-selected tooth surfaces, as follows: 0 = No debris/no calculus or stain present; 1 = Soft debris/supragingival calculus covering no more than 1/3 of the tooth surface; 2 = Soft debris/supragingival calculus covering more than 1/3, but not more than 2/3 of the tooth surface; and, 3 = Soft debris/supragingival calculus covering more than 2/3 of the exposed tooth surface. The six surfaces evaluated using the OHI-S were selected from four posterior and two anterior teeth (World Health Organization, 2013). The Simplified Oral Hygiene Index (OHI-S) dichotomized into poor and good (OHI-S ≥2 and <2) (García-Pérez et al., 2017); dental and medical visits ≤6 months (yes/no); and, the subject’s mother’s level of educational attainment (<9 years and ≥9 years). The variable of level of educational attainment was used to compare the results obtained for subjects whose mothers who had completed nine or more years of formal education with those whose mothers had completed less than nine years, which, in Mexico, corresponds to primary and secondary school combined (García-Pérez et al., 2021).

The household SES was calculated based on the presence of specific items in the household and the educational level of the heads of the family, in accordance with CONAPO classification criteria. Said criteria defines a *high income level* as a mean household purchasing power characterized by parents with permanent employment, high income levels, and a high level of educational attainment and corresponds to a group comprising professionals and businesspeople. A *middle income level* corresponds to the mean purchasing power of households characterized by parents with permanent employment, middle income levels, and a medium level of education. A *low income level* corresponds to larger families characterized by parents with little formal education and receiving a low level of income from temporary jobs in construction and other informal sectors (CONAPO, 2010; Molina-Frechero et al., 2017). All of the foregoing information was obtained *via* a questionnaire sent to the participants’ parents/guardians, with ten questionnaires excluded because they had not been filled out.

The DAI criteria were used to measure both the presence/absence of malocclusion and its severity. Comprising two components (aesthetic and dental), the DAI includes ten variables for dentofacial anomalies related to both clinical and aesthetic aspects: missing anterior teeth; incisal segment spacing; midline diastema; incisal segment crowding; largest anterior irregularity in the maxilla; largest anterior irregularity in the mandible; anterior maxillary overjet; anterior mandibular overjet; anterior open bite; and, anterior crossbite. The DAI component scores were multiplied by a specific weighting set out by the WHO, with a constant added in order to obtain a final DAI score for each child. Subsequently, the DAI score was classified into four categories of malocclusion: (DAI ≤25) normal or minor; (DAI 26–30) definite; (DAI 31–35) severe; and, (DAI ≥36) very severe (Jenny & Cons, 1996).

#### Outcome variable

The OHRQoL was evaluated using the Spanish version of the CPQ_8−10_ (Villanueva-Gutiérrez et al., 2019), which was designed exclusively for this age group and has been proven to be valid and reliable for use with Mexican children ( Del Carmen Aguilar-Díaz & Irigoyen-Camacho, 2011). The CPQ_8−10_ consists of 25 questions, giving a score ranging from 0 to 100, and four domains: oral symptoms; functional limitations; emotional well-being; and, social well-being. The higher the CPQ_8−10_ value, the greater the negative impact of the oral conditions on the child’s quality of life. The questionnaire also contains two questions requesting the child’s personal information (age and gender) and two global questions, one related to the child’s general perception of the state of their oral health and the other related to the extent to which the child’s oral/orofacial condition affects their general well-being. The higher the CPQ_8−10_ value, the greater the negative impact of the oral conditions on the child’s quality of life.

The questionnaire also contains two questions requesting the child’s personal information (age and sex) and two global questions, one related to the child’s general perception of the state of their oral health and the other related to the extent to which the child’s oral/orofacial condition affects their general well-being.

### Clinical oral examination

The clinical oral evaluations were conducted in selected schools by two dentists using dental mirrors and a WHO probe. The examination of the children’s oral cavity adhered to the corresponding infection control standards. Two examiners participated in the training and calibration exercise, which consisted of two steps (theoretical and clinical), using the DAI index and the OHI-S, while their inter and intra-examiner agreement for the 10 DAI conditions corresponded to a Cohen’s kappa coefficient of >90% and an OHI-S >0.80, respectively.

### Study size

Non-probability sampling was carried out for convenience at three schools selected in the northern, central, and southern sections of the study area, with all those pupils aged between 8–10 years invited to participate. The sample size of 480 children was designed to detect an odds ratio of (OR) = 2.0, with 80% power and an alpha of 0.05, and a probability of 0.30 for malocclusion, considering the probability that malocclusion has a negative impact on the subject’s OHRQoL.

### Statistical analysis

A bivariate analysis was performed using Pearson’s chi-square (qualitative variables: sex, Toothbrushing frequency, OHI-S, Mother’s level of education, Dental visits, medical visits. SES), the Kruskal-Wallis, and Wilcoxon’s rank (quantitative variables, non-normal distribution) tests.

Five Poisson regression models with robust variance were used to ascertain the association between the OHRQoL score and the independent variables, with the first association determined between the total CPQ_8−10_ score and the independent variables. The following four models were constructed to determine the association between the four domains of the CPQ_8−10_ (oral symptoms, functional limitations, emotional well-being, and social well-being) and the independent variables. Overall, the CPQ_8−10_ and specific domain scores were compared in terms of the rate ratios (RRs) and respective 95% confidence intervals (95% CIs) of interest as well as the confounding variables, with values of *p* ≤ 0.05 considered statistically significant. Data analysis was performed using the Stata 15 program.

## Results

Table 1 presents the data generated by the descriptive and bivariate analysis conducted on the 480 (100%) eight-to-ten-year-old child subjects, whose mean age was 9.2 (±0.75) years, while the percentages of boys and girls examined were 246 (50.6%) and 237 (49.4%), respectively. Our findings reveal that 281 (58.5%) of the children brushed their teeth once per day or less, 266 (55.4%) of them had poor oral hygiene, 301 (62.7%) had not visited a dentist in the last six months, and 74 (15.4%) had not visited a doctor in the last six months, while, in terms of their household SES, 218 (45.4%) presented a low income.

**Table 1:** Bivariate analysis of malocclusion and variables of 480 schoolchildren from 8 to 10 years from Mexico.

	Normal *n* = 99	Definite *n* = 150	Severe/ Very severe *n* = 231	Value *p*
Age^a^	9.3 (±0.74)	9.2 (±0.75)	9.1 (±0.75)	0.043
Sex				
Male	48 (48.5)	70 (46.7)	125 (54.1)	0.325
Female	51 (51.5)	80 (53.3)	106 (45.9)	
Toothbrushing frequency				
≥ 2 times a day	43 (43.4)	59 (39.3)	97 (42.0)	0.792
<2 times a day	56 (56.6)	91 (60.7)	134 (58.0)	
Oral hygiene (OHI–S)				
Good hygiene	50 (50.5)	63 (42.0)	101 (43.7)	0.391
Poor hygiene	49 (49.5)	87 (58.0)	130 (56.3)	
Mother’s level of education				
<9 years	53 (53.5)	68 (45.3)	119 (51.5)	0.365
≥ 9 years	46 (46.5)	82 (54.7)	112 (48.5)	
Dental visits ≤6 months				
No	56 (56.6)	87 (58.0)	158 (68.4)	0.045
Yes	43 (43.4)	63 (42.0)	73 (31.6)	
Medical visits ≤6 months				
No	15 (15.2)	18 (12.0)	41 (17.7)	0.315
Yes	84 (84.8)	132 (88.0)	190 (82.3)	
Socioeconomic status (SES)				
High–income	34 (34.3)	31 (20.7)	45 (19.5)	0.050
Middle–income	27 (27.3)	49 (32.7)	76 (32.9)	
Low–income	38 (38.4)	70 (46.6)	110 (47.6)	

Notes.

a Kruskal–Wallis test.

b Chi-square test.

Our findings show that 381 (79.4%) of participants had some type of malocclusion in their mixed dentition, presenting 150 (31.3%) definite, 123 (25.6%) severe, and 108 (22.5%) very severe malocclusion. The bivariate analysis conducted found no statistical significance for an association between sex and malocclusion severity; however, age (*p* = 0.043), a low dental visit frequency (*p* = 0.045), and a low income level (SES) (*p* = 0.050) all showed a statistically significant association with malocclusion severity (Table 1). Table 2 presents the distribution of the participants according to the DAI components and their need for orthodontic treatment.

**Table 2:** Distribution of sample according to type of malocclusion, treatment need, and dentition status (*n* = 480) in schoolchildren from 8 to 10 years from Mexico.

	*n*	%
Missing anterior teeth		
Maxillary dental arch	134	27.9
Mandibular dental arch	85	17.7
Anterior crowding		
None	125	26.0
One or two segments	355	74.0
Incisal segment spacing		
None	308	64.2
One or two segments	172	35.8
Diastema		
<2mm	291	60.6
≥ 2 mm	189	39.4
Largest anterior irregularity in the maxilla		
<2mm	278	57.9
≥ 2 mm	202	42.1
Largest anterior irregularity in the mandible		
<2mm	348	72.5
≥ 2 mm	132	27.5
Anterior maxillary overjet		
<4 mm	229	47.7
≥4 mm	251	52.3
Anterior mandibular overjet		
<4 mm	362	75.4
≥4 mm	118	24.6
Anterior open bite		
<2mm	459	95.6
≥ 2 mm	21	4.4
Anterior crossbite		
Absent	261	54.4
Present	219	45.6
Orthodontic treatment need		
None	99	20.6
Elective	150	31.3
Highly desirable	123	25.6
Fundamental	108	22.5

Based on their general perception of the state of their oral health, 17.9% of the participants rated their oral health as very good, 29.0% as good, 43.5% as regular, and 9.6% as poor. With regard to the extent to which the child’s oral/orofacial condition affects their general well-being, 52.7% of the children reported experiencing a negative impact on their quality of life due to the condition of their mouth, while 47.3% indicated no impact.

When the means for the four CPQ_8−10_ domains and malocclusion severity were compared, significant differences were found for the following: overall score (*p* < 0.001); oral symptoms (*p* < 0.001); functional limitations (*p* < 0.001); emotional well-being (*p* < 0.001); and, social well-being (*p* < 0.001). Moreover, the following significant differences were found between socioeconomic status and the four CPQ_8−10_ (total score CPQ_8−10_ –*p* = 0.001) domains: oral symptoms (*p* = 0.013); functional limitations (*p* = 0.006); emotional well-being (*p* = 0.006); and, social well-being (*p* = 0.004). Table 3 presents the total distribution by CPQ_8−10_ domain, with the malocclusion severity levels and the association between the independent variables demonstrating that most of the children with severe/very severe malocclusion and who resided in a middle or low income household presented a higher score for the CPQ_8−10_ and its four domains. Poor oral hygiene and low dental visit frequency had a negative impact on three of the CPQ_8−10_ domains (oral symptoms, emotional well-being, and social well-being).

**Table 3:** Overall CPQ_8−10_ score and subscales by Malocclusion severity, socioeconomic status, oral hygiene habits in schoolchildren 8 to 10 years (*n* = 480).

		Total score CPQ		Oral symptoms		Functional limitation		Emotional well–being		Social well–being	
		mean (SD)	median	mean (SD)	median	mean (SD)	median	mean (SD)	median	mean (SD)	median
Severity malocclusion	Normal	11.3 (7.6)^**^	10^*^	3.0 (2.4)^**^	2^*^	2.4 (2.8)^**^	1^*^	2.4 (2.9)^**^	2^*^	3.3 (3.4)^**^	2^*^
	Definite	27.1 (17.6)	24	7.4 (5.3)	6.5	4.1 (4.4)	2	4.4 (4.8)	3	11.0 (9.2)	10
	Severe/ Very severe	36.7 (23.2)	32	8.8 (5.7)	9	6.8 (5.3)	7	6.8 (5.8)	6	14.3 (11.7)	11
											
Socioeconomic status	High	22.4 (20.2)^*^	15^*^	5.7 (4.8)^*^	4^*^	4.3 (4.1)	3	3.8 (4.2)^*^	2^*^	8.6 (10.6)^*^	5
	middle	28.8 (20.6)	26	7.5 (5.3)	8	4.7 (4.8)	2.5	5.1 (5.6)	3	11.6 (10.2)	10
	Low	31.2 (22.1)	25	7.7 (5.9)	6	5.7 (5.3)	3.5	5.9 (5.4)	4	11.8 (10.8)	10
OHI–S	Good hygiene	24.8 (21.0)^**^	17^*^	6.4 (5.4)^**^	5^*^	4.7 (4.9)	2	4.2 (4.8)^**^	2^*^	9.5 (10.4)^**^	7^*^
	Poor hygiene	31.3 (21.4)	27	7.8 (5.5)	7	5.3 (5.0)	3	5.9 (5.6)	4	12.2 (10.7)	10
Toothbrushing frequency	≥ 2 times a day	28.2 (21.7)	22	7.3 (5.6)	6	5.3 (5.2)	3	5.1 (5.1)	3	10.5 (10.8)	8
	<2 times a day	28.6 (21.3)	23	7.1 (5.5)	6	4.9 (4.7)	3	5.2 (5.4)	3	11.4 (10.5)	10
											
Dental visits	<6 months	25.3 (21.6)^**^	18^*^	6.5 (5.2)^*^	6^*^	4.6 (4.9)	3	4.3 (5.0)^*^	2^*^	9.8 (10.9)^*^	7^*^
	≥ 6 months	30.3 (21.2)	26	7.6 (5.7)	7	5.3 (4.9)	3	5.6 (5.4)	4	11.7 (10.4)	10

Notes.

* *p* < 0.05.

** *p* ≤ 0.001.

In terms of malocclusion severity, the Poisson model showed that children with definite or severe/very severe malocclusion reported a significantly greater negative impact in terms of their overall OHRQoL score and each domain of the CPQ_8−10_ (Table 4). Children from a low-income household experienced a higher negative impact than children from a high-income household, for both their overall OHRQoL score and each domain of the CPQ_8−10_ (Table 4). Furthermore, poor oral hygiene, a low frequency of dental visits (≤6 months), and the participant’s mother’s level of educational attainment (<9 years) had a negative impact on OHRQoL, in terms of both the overall score and the CPQ_8−10_ domains. Finally, the interaction between the participant’s mother’s level of educational attainment and the household’s SES was evaluated, with no statistically significant differences found. Poisson regression analysis did not detect multicollinearity among the independent variables.

**Table 4:** Adjusted rate ratio (RR) from Poisson regression analysis for the oral health-related quality of life (OHRQoL) and malocclusion severity, socioeconomic status and confounding variables in schoolchildren 8–10 years of age (*n* = 480).

		Total score CPQ	Oral symptoms	Functional limitation	Emotional well–being	Social well–being
		Robust Rate Ratio (RR) (95% Confidence Interval)				
Sex	Boys	1.00	1.00	1.00	1.00	1.00
	Girls	0.98 (0.95–1.02) *p* = 0.525	0.98 (0.92–1.05) *p* = 0.679	1.01 (0.93–1.09) *p* = 0.781	1.02 (0.95–1.11) *p* = 0.476	0.96 (0.91–1.01) 0=0.169
OHI–S	Good hygiene	1.00	1.00	1.00	1.00	1.00
	Poor hygiene	1.21 (1.16–1.25) *p* < 0.001	1.18 (1.10–1.26) *p* < 0.001	1.09 (1.00–1.18) *p* = 0.032	1.34 (1.24–1.46) *p* < 0.001	1.22 (1.15–1.29) *p* < 0.001
Toothbrushing frequency	≥ 2 times a day	1.00	1.00	1.00	1.00	1.00
	<2 times a day	1.01 (0.98–1.05) *p* = 0.275	0.97 (0.91—-1.04) *p* = 0.507	0.93 (0.85–1.00) *p* = 0.085	1.02 (0.94–1.11) *p* = 0.506	1.08 (1.03–1.15) *p* = 0.003
Dental visits ≤ 6 months	Yes	1.00	1.00	1.00	1.00	1.00
	No	1.10 (1.06–1.14) *p* < 0.001	1.10 (1.02–1.18) *p* = 0.009	1.01 (0.93–1.11) *p* = 0.660	1.16 (1.06–1.27) *p* = 0.001	1.11 (1.04–1.18) *p* < 0.001
Mother level of education	≥ 9 years	1.00	1.00	1.00	1.00	1.00
	<9 years	1.11 (1.08–1.15) *p* < 0.001	1.14 (1.07–1.22) *p* < 0.001	1.01 (0.93–1.09) *p* = 0.736	1.12 (1.04–1.22) *p* = 0.003	1.14 (1.08–1.20) *p* < 0.001
Socioeconomic status	High–income	1.00	1.00	1.00	1.00	1.00
	Middle–income	1.16 (1.11–1.22) *p* < 0.001	1.19 (1.08–1.32) *p* < 0.001	0.99 (0.88–1.11) *p* = 0.901	1.22 (1.08–1.37) *p* = 0.001	1.20 (1.11–1.30) *p* < 0.001
	Low–income	1.24 (1.19–1.30) *p* < 0.001	1.22 (1.11–1.33) *p* < 0.001	1.20 (1.08–1.34) *p* = 0.001	1.38 (1.24–1.55) *p* < 0.001	1.22 (1.13–1.31) *p* < 0.001
Severity Malocclusion	Normal	1.00	1.00	1.00	1.00	1.00
	Definite	2.31 (2.16–2.47) *p* < 0.001	2.38 (2.09–2.70) *p* < 0.001	1.65 (1.42–1.92) *p* < 0.001	1.71 (1.48–1.99) *p* < 0.001	3.16 (2.80–3.55) *p* < 0.001
	Severe/ Very severe	3.09 (2.90–3.29) *p* < 0.001	2.78 (2.46–3.14) *p* < 0.001	2.72 (2.37–3.11) *p* < 0.001	2.59 (2.25–2.97) *p* < 0.001	3.99 (3.57–4.47) *p* < 0.001

## Discussion

The present study found that its eight-to-ten-year-old subjects with severe/very severe malocclusion in their mixed dentition and living in a household with a low income level experienced a negative impact both on their overall OHRQoL score and in each domain of the CPQ_8−10_. Little research is reported in the literature that has evaluated the impact of malocclusion severity and SES on quality of life during the mixed dentition stage. Both Vedovello et al. (2016) and Simões et al. (2017) found that the presence of severe/very severe malocclusion and a low household SES had a negative impact on the participants’ OHRQoL. The present study found that malocclusion severity had a greater negative impact in oral, functional, emotional, and social terms in the children sampled.

Contradictory results have been reported in terms of malocclusion severity, with a study conducted on eight-to-ten-year-old children with mixed dentition and using the CPQ_8−10_ finding no differences between the severity revealed by the DAI and the OHRQoL (*p* > 0.05); moreover, it also found no relationship between household SES and malocclusion (Piassi et al., 2019). Martins-Júnior, Marques & Ramos-Jorge (2012) in a study conducted on eight-to-ten-year-old participants, observed that malocclusion severity is related to a greater negative impact on the participants’ OHRQoL.

The mixed dentition stage is a period of prolonged development occurring between the ages of six and 12 and is susceptible to localized factors that, if undetected, may result in serious malocclusion problems (dentoalveolar anterior crossbite, ectopic eruption of permanent incisors and/or first permanent molars, posterior crossbite, and open bite, among others). Therefore, the identification of the changes produced during dental eruption, the early diagnosis of malocclusions, and the importance of taking a preventative approach during this transitional process all indicate the importance of the present research, especially for this age group.

Research has been published on the aesthetic and functional impact of malocclusion on OHRQoL in children between the ages of eight and ten years, reporting that children with malocclusions presented a greater negative day-to-day impact in oral, functional, emotional, and social terms than those children without malocclusion (Guimarães, Jorge & Fontes, 2018). The present study found that definite and severe/very severe malocclusion had an impact on the OHRQoL, revealing an association between malocclusion and both emotional and social well-being, as well as oral and functional limitations, thus leaving these children with low self-esteem (Agou et al., 2008).

Appearance is fundamental for social relationships and interaction in the course of people’s day-to-day activities, with, from an early age, children beginning to compare their physical and personality characteristics with those of other children. From between six and ten years old, a child will develop their capacity to make judgments over their appearance, thoughts, and emotions; furthermore, at this age, health-associated aesthetics begins to be incorporated into the mind of the child and, thus, becomes integrated into their concept of self-esteem (Rebok, Riley & Forrest, 2001). Similarly, an understanding of these concepts also seems to be affected by the sex of the child, demonstrating that girls sustain a greater negative impact on their OHRQoL (Calis et al., 2009), although the present study did not find significant OHRQoL differences by sex.

As mentioned above, SES is another important factor found to be related to the relationship between malocclusion and OHRQoL. The present study found that a low household income and the presence of definite and severe/very severe malocclusion were related to a poor OHRQoL score. Prior research has demonstrated that the negative impact of oral conditions on OHRQoL varies in relation to the participant’s household SES (Paula et al., 2015). Ravaghi et al. (2019) note that the interrelationship among oral conditions, SES, and OHRQoL may be explained by direct and indirect mediating relationships. Among the direct mediating relationships are access to services and the participant’s mother’s level of educational attainment, with the present study finding that a low number of visits to the dentist in the first six months and a mother with <9 years of schooling had a negative impact on the child’s OHRQoL. Moreover, a low number of visits to the dentist was found to be related to the severity of the malocclusion (*p* = 0.045).

The foregoing leads to the conclusion that the participant’s mother’s level of educational attainment, in terms of both learning and knowledge, plays an important role in the taking of health-related decisions (Medina-Solís et al., 2006). Moreover, psychological factors, such as a sense of control, perceived stress, and satisfaction with one’s day-to-day life, affect OHRQoL, while those participants from disadvantaged populations with a lack of psychosocial resources present low expectations with regard to their appearance and, thus, may be affected by malocclusion (Ravaghi et al., 2019).

Poor hygiene was negatively associated with OHRQoL, while the prevalence of severe/very severe malocclusion was higher in participants with poor oral hygiene (56.3%), although the present study did not find differences in terms of this association. Prior studies have established that the presence of crowding has implications in terms of poor oral hygiene (Ngom et al., 2006), given that the displacement of the contact points and the disharmony of an unaligned dental arch promotes the retention of plaque. The foregoing has established the importance of the timely treatment of malocclusions, with the objective of ensuring optimal periodontal conditions.

One of the limitations of the present study is its cross-sectional design, given that it is not possible to determine causal associations and that bias may also be present. Another limitation is that various studies use various indicators to evaluate SES, which may, thus, reduce the comparability of the present study. Finally, the non-randomization of the selection process for those children who would be invited to participate is also a limitation of the research presented here.

## Conclusion

• Definite and severe/very severe malocclusion and a low SES have a negative impact on the four OHRQoL domains of oral symptoms, functional limitations, social well-being, and emotional well-being.
• The OHRQoL assessment may vary in terms of the socioeconomic levels of the subjects’ households that exert possible consequences on their oral health.

##  Supplemental Information

10.7717/peerj.12062/supp-1 Supplemental Information 1 Stata data of malocclusion in mixed dentition and OHRQoL of eight-to-ten- year-old

10.7717/peerj.12062/supp-2 Supplemental Information 2 Checklist STROBE STROBE Statement—Checklist of items that should be included in reports of ***cross-sectional studies***

## Additional Information and Declarations

### Data Availability

The following information was supplied regarding data availability: Raw data are available in the Supplementary File.
